# Nanomaterials for Periodontal Tissue Regeneration: Progress, Challenges and Future Perspectives

**DOI:** 10.3390/jfb14060290

**Published:** 2023-05-24

**Authors:** Chen Zong, Annelies Bronckaers, Guy Willems, Hong He, Maria Cadenas de Llano-Pérula

**Affiliations:** 1 Department of Oral Health Sciences-Orthodontics, University of Leuven (KU Leuven) and Dentistry, University Hospitals Leuven, 3000 Leuven, Belgium; 2 Biomedical Research Institute, Faculty of Life Sciences, University of Hasselt, 3590 Diepenbeek, Belgium; 3 The State Key Laboratory Breeding Base of Basic Science of Stomatology (Hubei-MOST) and Key Laboratory of Oral Biomedicine Ministry of Education, School & Hospital of Stomatology, Wuhan University, Wuhan 430079, China; 4 Department of Orthodontics, School & Hospital of Stomatology, Wuhan University, Wuhan 430079, China; 5 Center for Dentofacial Development and Sleep Medicine, School & Hospital of Stomatology, Wuhan University, Wuhan 430079, China

**Keywords:** nanomaterials, periodontal regeneration, alveolar bone, periodontal ligament, cementum, gingiva, toxicity

## Abstract

Bioactive nanomaterials are increasingly being applied in oral health research. Specifically, they have shown great potential for periodontal tissue regeneration and have substantially improved oral health in translational and clinical applications. However, their limitations and side effects still need to be explored and elucidated. This article aims to review the recent advancements in nanomaterials applied for periodontal tissue regeneration and to discuss future research directions in this field, especially focusing on research using nanomaterials to improve oral health. The biomimetic and physiochemical properties of nanomaterials such as metals and polymer composites are described in detail, including their effects on the regeneration of alveolar bone, periodontal ligament, cementum and gingiva. Finally, the biomedical safety issues of their application as regenerative materials are updated, with a discussion about their complications and future perspectives. Although the applications of bioactive nanomaterials in the oral cavity are still at an initial stage, and pose numerous challenges, recent research suggests that they are a promising alternative in periodontal tissue regeneration.

## 1. Introduction

The oral cavity plays a crucial role in essential body functions such as mastication, speech or deglutition, and its influence on facial aesthetics, especially of teeth, is undeniable. Periodontal tissue, including the alveolar bone, periodontal ligament (PDL), cementum and gingiva, is essential to maintain the integrity and stability of the teeth, absorb the chewing forces, and protect against bacterial invasion and infection. There are a variety of factors causing periodontal tissue defects and threatening the patients’ quality of life, such as caries, periodontitis, tumors, cysts and trauma [1]. Due to the complex functions of the oral tissue and the unique characteristics of the oral environment, it has always been a great challenge to reconstruct the periodontal tissue and restore its physical function.

For periodontal tissue reconstruction, traditional techniques based on allogenic grafts replacing the missing or damaged tissue from living donors or even cadavers are still used in dental and other medical fields [2]. For example, autologous and allogenic alveolar bone grafts are currently considered a gold standard to overcome bone atrophy, although in clinical settings the best results seem to be obtained with autologous bone [3]. However, these methods encounter limitations such as limited supply of graft tissue, donor site morbidity, graft failure, immunological rejection and lengthy hospitalization periods [4]. In addition, these techniques exhibit great difficulty in regenerating the cementum–ligament–bone complex. Furthermore, traditional dental materials such as composites and cement in macro and micro sizes are also widely applied in clinics. Despite their low cost, easy application and good biocompatibility, these materials also present several complications, such as degradation [5], cure shrinkage [6], stress fatigue [7], marginal microleakage [8] and high susceptibility to microbial adhesion. Thus, there is a critical need for alternative techniques and materials.

In this respect, nanotechnology can provide an innovative alternative. Nanomaterials, in the range of 1–100 nanometers, have gained significant attention in regeneration medicine due to their unique optical, mechanical, magnetic, electronic and catalytic properties [9], which explain their high biocompatibility, permeability, tunability and immune evasion capability. Hence, they exhibit tremendous potential in tissue engineering [10], as anti-bacterial agents [11], for drug delivery [12], tissue repair [13] and functional imaging (such as MRI and CT) [14]. Recently, various types of nanomaterials, such as nanoparticles, nanocapsules, nanocomposites, nanofibers, nanotubes and nanosheets, have achieved satisfying outcomes and could therefore be used to reconstruct structures and restore the functions of oral tissues [15].

However, nanotechnology and nanomaterials also face great challenges that prevent them from advancing from the bench to the clinic. Firstly, there is a shortage of established protocols that allow their construction with the desired composition, structure parameters and physiochemical properties. It is difficult to modify or improve the behavior or performance of nanosystems in vivo because of the limited development of their surface chemistry [16]. Second, the biosafety issues and adverse events of nanomaterials remain concerning. For instance, in vivo application of nanomaterials could induce immunologic reactions [17]. Moreover, nanomaterials have shown high permeability [18], and therefore their cytotoxicity can significantly increase as they can penetrate through physiological barriers and may accumulate in nontarget tissues [19]. Third, it is very difficult to control their biodegradation. Their biodistribution and pharmacokinetics largely depend on the size, shape and surface chemistry and homogeneous production of nanomaterials is still a challenge [20]. Moreover, when nanomaterials are applied for the local delivery of drugs, it is essential to control their biodegradation rate [21]. Although many papers emphasize their promising perspective in tissue regeneration, these challenges are rarely discussed.

**Figure 1:**
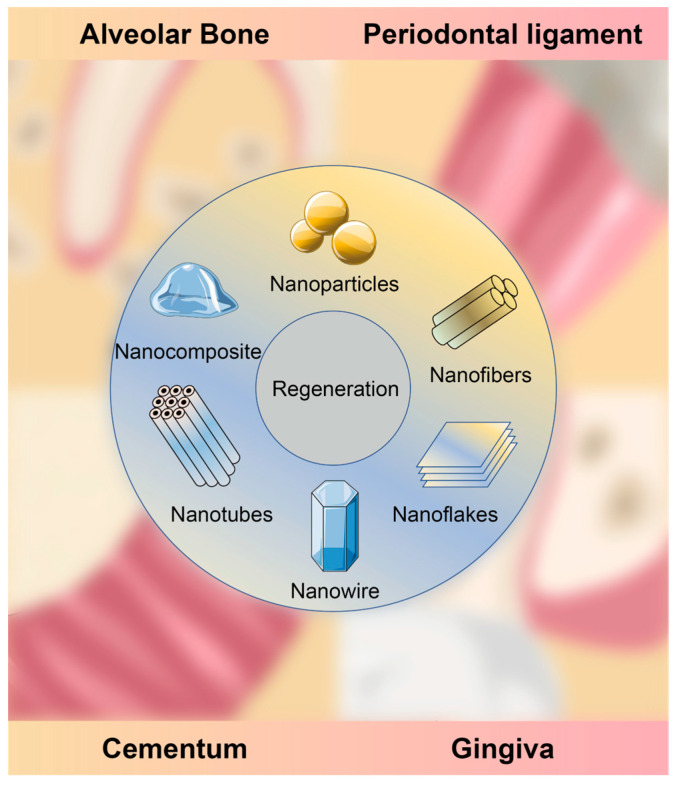
Periodontal tissue regeneration with nanomaterials.

This review focuses on the most recent publications regarding periodontal tissue regeneration with nanotechnology and nanomaterials (Figure 1). First, the current designs, structures and functions of nanomaterials are introduced and discussed. Secondly, the related factors that may interact with the behaviors and bioactivities of nanomaterials are summarized and elucidated. Lastly, the potential complications of nanomaterials are presented and some remarks for future research are proposed in order to overcome the current limitations.

## 2. Materials and Methods

### 2.1. Literature Search Strategy

An electronic literature search was conducted on PubMed using the following combination of search terms and Boolean operators: “((alveolar bone regeneration) OR (cementum regeneration) OR (gingiva regeneration) OR (periodontal ligament regeneration)) AND (nanomaterials OR nanotechnology) NOT review.” to identify articles published in the last 5 years (between January 2017 and February 2023). No filters were activated and similar additional searches were performed on Embase and Cochrane Library.

### 2.2. Literature Screening and Selection Criteria

Literature screening and selection were carried out by one reviewer. The titles and abstracts of the publications identified by the different databases were screened and the reference lists of critical articles were additionally and hand search for relevant articles. The full text was examined at the second stage to determine whether it matched the selection criteria.

The inclusion criteria were as follows:

(1) The reports on nanomaterials were not older than 5 years (published before 2017).
(2) The nanomaterials should be proposed to promote periodontal tissue regeneration, including alveolar bone, PDL, cementum and gingiva.
(3) The literature should involve research in not only physiochemistry but also biomedicine, for which the nanomaterials were tested in cells and/or animal models.

The exclusion criteria were as follows:

(1) The materials used for periodontal tissue regeneration are not nanomaterials.
(2) The nanomaterials were not designed for periodontal tissue regeneration but rather for osseointegration of dental implants.
(3) Case reports with a sample size smaller than three subjects.

### 2.3. Data Management

The following data were extracted from the included articles in an Excel Table: application, nanomaterials, morphology, in vitro experiments, in vivo experiments, outcomes and references. Articles were sorted according to the type of tissue they intended to regenerate (alveolar bone, PDL, cementum, gingiva).

## 3. Results

### Literature Search

**Figure 2:**
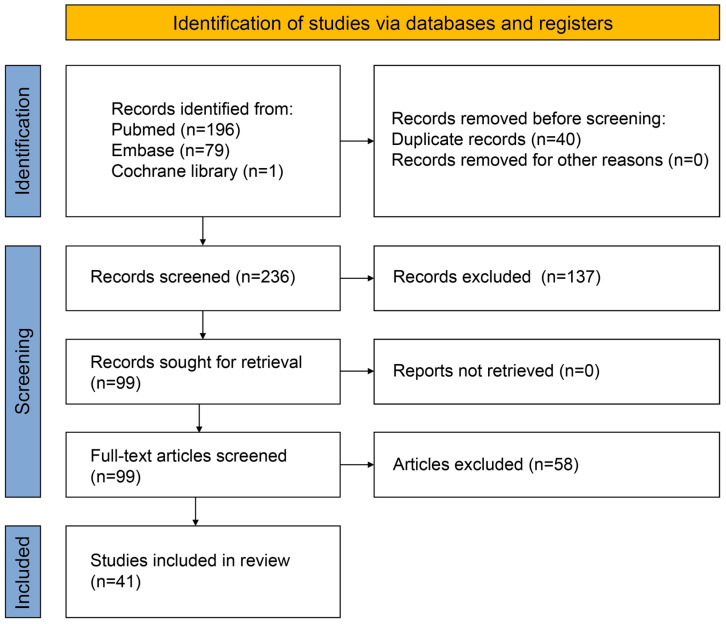
Flowchart of the retrieved and selected literature.

A total of 276 literature studies were acquired from the three databases, from which 99 were selected and read as full text after the screening. Forty-one studies met the eligibility criteria and were included in this study, as shown in Figure 2.

## 4. Discussion

### 4.1. Alveolar Bone Regeneration

The alveolar bone is a specific type of bone tissue that forms the sockets where the teeth are supported and anchored to the jaws. The alveolar bone is constantly remodeling itself in response to various factors, such as chewing forces and hormonal changes. Under physiological conditions, this process involves the breakdown and replacement of old bone tissue by new bone tissue, which helps to maintain the integrity and strength of the bone. However, under pathological conditions, the breakdown of alveolar bone is spontaneously irreversible and can lead to tooth mobility and loss [22]. Most of the studies involving periodontal tissue regeneration focus on alveolar bone regeneration. Various techniques have been proposed to guide or control bone regeneration, such as bone grafting [23], guided tissue regeneration (GTR) [24] and platelet-rich plasma (PRP) therapy [25]. Although effective to a certain level, these techniques lack in repeatability and do not completely reconstruct the original periodontium [26,27]. Recently, the application of nanomaterials smaller than 100 nm on the above techniques has shown promising results, since they have multiple advantages over traditional materials, such as versatility, biocompatibility, enhanced cellular interactions, improved tissue integration, controlled drug release and mechanical strength [28,29,30].

Among the included studies, the nanomaterials applied in alveolar bone regeneration are listed in Table 1. *Nano-hydroxyapatite* (nHA) [31,32,33,34,35,36,37,38,39] and *collagen* [31,39,40,41,42,43] are the most commonly investigated biomaterials for alveolar bone regeneration. Hydroxyapatite is a naturally occurring mineral that is the main component of bone tissue, and nHA has a higher surface area-to-volume ratio compared to conventional hydroxyapatite, making it more suitable as a substitute for bone graft [44]. Collagen is a crucial component of the extracellular matrix in many tissues, including alveolar bone. It provides structural support, enables cell–biomaterial interaction and increases cell adhesion, which helps regulate cell behavior, making it an attractive biomaterial for alveolar bone regeneration [45]. Both nHA and collagen have excellent osteoconductive properties, which can support the formation of new bone tissue by serving as a scaffold for bone growth, with good biocompatibility and mechanical properties.

*Poly(lactic-co-glycolic acid)* (PLGA) [41,42,46,47,48,49] and *polycaprolactone* (PCL) [38,46,50,51,52] are biodegradable polymers that have been widely studied as materials for nanoparticles but also in other applications in tissue engineering and regenerative medicine. Both PLGA and PCL degrade over time into natural compounds that can be metabolized and eliminated through normal metabolic pathways. This facilitates their application in bone grafting, guided tissue regeneration and especially drug delivery. When engineered to release therapeutic agents, such as growth factors or antibiotics, they can provide sustained long-term therapeutic effects that can promote bone regeneration and prevent inflammation [53]. However, PLGA and PCL also present shortcomings. For example, the mechanical strength of PLGA alone is inadequate for bone tissue regeneration, so PLGA often needs to be incorporated with other ceramic nanoparticles such as nHA, PCL and flourhydroxyapatite [54]. Moreover, although PCL displays adequate cell adhesion and good mechanical strength, its slow biodegradation, which takes more than 2 years, greatly exceeds the time required for new tissue formation [51], which can negatively impact the bone tissue regeneration process [55].

*Gold nanoparticles* (AuNPs) have attracted much attention as multifunctional contrast agents for computerized tomography (CT) due to their chemical inertness, versatile surface functionalization and biocompatibility, high radiopacity and low cytotoxicity [56,57]. In addition, in vivo cell labeling and tracking using AuNPs with CT have become a cost-effective and time-efficient approach [58]. Among the included studies, AuNPs have also been shown to have high potential in alveolar bone regeneration [13,59,60]. Besides the advantages mentioned above, AuNPs can promote the proliferation and differentiation of bone-forming cells, such as osteoblasts and mesenchymal stem cells [61,62]. Furthermore, AuNCs exhibit antibacterial properties, which can be useful in preventing infection during the bone regeneration process [63]. Finally, their property of X-ray attenuation can be useful to distinguish the transplant from the host tissue, monitor the regeneration process and evaluate the efficacy of the treatment.

*Bone morphogenic protein 2* (BMP-2) is one of the most popular drugs loaded in nanomaterials for alveolar bone regeneration [42,50,64,65]. BMP-2, a member of the transforming growth factor-β superfamily, has been shown to successfully promote alveolar bone regeneration by enhancing vertical bone height in animal studies [66,67]. However, it is important to note that BMP-2 is a potent growth factor and should be used with caution since it can lead to complications such as excessive bone growth, inflammation, fatty tissue formation and deteriorated bone quality [68].

The number of studies on alveolar bone regeneration greatly exceeds those focused on PDL, cementum or gingiva regeneration. However, it should be noted that periodontal tissue defects are usually accompanied by an inflammatory microenvironment, so alveolar bone regeneration has higher requirements regarding anti-inflammatory and immune regulation, which differs from regeneration of other bony structures [69]. This might be one of the most important limitations of studies applying an animal model of surgically inflicted bone defects since the surgical insult induces acute inflammation, in contrast to, i.e., a ligature-induced periodontitis model, which causes the chronic inflammation characteristic of human periodontitis [70]. The effect of nanomaterials in alveolar bone regeneration should be tested under particular conditions similar to clinical conditions to facilitate their translational application from bench to bedside.

**Table 1:** Nanomaterials used for alveolar bone regeneration.

Application	Nanomaterials	Morphology	*In Vitro* Experiments	*In Vivo* Experiments	Outcomes	References
Alveolar bone regeneration	SP600125, bone morphogenic protein 2 (BMP-2)	nanofiber	Beagle PDLCs and bone marrow stem cells (BMSCs)	Beagle model of mandible class II furcation defect	suppress the expression of pro-inflammatory factors and recover bone defects covering the periodontitis site within 2 month	Liu et al., 2020 [64]
	Electrospun fish collagen/bioactive glass/chitosan (Col/BG/CS)	nanofiber	hPDLCs	Beagle model of mandibular furcation defect	enhance the cell viability and osteogenic gene expression, increase the expression of RUNX-2 and OPN protein, and promote bone regeneration	Zhou et al., 2017 [40]
	Ferroelectric BaTiO_3_/poly(vinylidene fluoride-trifluoroethylene)	nanocomposite	N/A	Mini-pig model of bone defect	prevent the vertical and horizontal dimension resorption of the alveolar ridge, promote buccal alveolar bone regeneration and maturation	Li et al., 2023 [71]
	Nano β-tricalcium phosphate/chitosan /glycerophosphate/glyoxal hydrogel	nanoparticle	Wish normal cells, hepatocellular carcinoma and breast cancer cell lines	Mongrel dog model of mandible bone defects	promote new bone in infected teeth	Abdel-Fattah et al., 2017 [72]
	Poly(L-lactide-co-D,L-lactide) encapsulating platelet-derived growth factor or metronidazole	nanofiber	MSCs line	Murine model of dentoalveolar defect	show high biocompatibility, facilitate wound healing and enhance alveolar ridge regeneration	Ho et al., 2017 [73]
	Lysophosphatidic acid, zinc oxide, poly(lactic-co-glycolic acid) (PLGA)/PCL and deferoxamine	nanofiber	Murine calvarial osteoblast cell line (MC3T3-E1) and Human umbilical vein endothelial cells	Murine model of maxillary alveolar bone defect, rat model of mandibular fenestration	exhibit remarkable biocompatibility and osteogenesis, antibacterial activity, neovascularization and new bone formation	Xing et al., 2022 [46]
	PCL biomembranes, BMP-2 and ibuprofen	nanoreservoir	hBMSCs	Murine model of maxillary alveolar bone lesion	regenerate maxillary bone for periods between 90 and 150 days after implantation	Stutz et al., 2020 [50]
	Autologous BMMNCs loaded collagen sponge with nano-HA and autologous platelet-rich fibrin	nanoparticle	N/A	Patients with unilateral alveolar cleft defects	exhibit less complications and better tissue healing. 90% of the cases exhibit complete alveolar bone union	Al-Ahmady et al., 2018 [31]
	silica coated nanoHA-gelatin reinforced with electrospun poly(L-lactic acid) fibres	nanofiber	N/A	Rabbit model of critical alveolar defects	promote bone formation in load bearing mandibular region	Manju et al., 2018 [32]
	Metformin hydrochloride, citric acid	carbon dots	Rat BMSCs	Rat model of ligature-induced periodontitis	promote BMSCs osteogenesis under normal and inflammatory conditions, and regenerate the lost alveolar bone in rats	Ren et al., 2021 [74]
	Gold nanoparticles, adenovirus-mediated human β-defensin 3 gene	nanoparticle	hPDLCs	Rat model of ligature-induced periodontitis	promote hPDLCs osteogenic differentiation and periodontal regeneration, via the p38 MAPK pathway	Li et al., 2021 [59]
	CaCO_3_/*miR-200c*	nanoparticle	Human embryonic palate mesenchymal cells	Rat model of alveolar defects	increase bone formation in rat alveolar bone defects	Remy et al., 2022 [75]
	CaP@P-fiber	nanofiber	Rat BMSCs and murine-derived macrophage cell line	Rat model of artificial alveolar bone defect	enhance the osteo-immunomodulatory and osteo-inductive functions, result in an excellent bone repair	He et al., 2022 [76]
	Polydopamine structure, bone-forming peptide-1, ascular endothelial growth factor-mimicking peptide	nanocomposite	hPDLSCs	Rat model of cranial defect	boost the proliferation and osteogenic differentiation of PDLSCs with improved cytocompatibility, regenerate of periodontal bone dramatically	Xiang et al., 2020 [77]
	PLGA-collagen-gelatin	nanofiber	Murine fibroblasts	Rat model of critical maxillary alveolar bone defect	reveal ~3 times greater new bone volume and bone mineral density compared to the unfilled control defects over 4 weeks	Boda et al., 2019 [41]
	gelatin/nano-HA/metformin	nanocomposite	Human MSCs	Rat model of critical maxillary alveolar bone defect	show bone regenerations with greater alveolar ridge preservation, and bone formation with less connective tissue and residual scaffold	Fang et al., 2022 [33]
	Alendronate, heptaglutamate conjugated BMP-2 mimicking peptide, PLGA-collagen-gelatin	nanofiber	N/A	Rat model of critical maxillary alveolar bone defect	elevate new bone volume fraction and bone mineral density	Boda et al., 2020 [42]
	Tetrahedral framework nucleic acids	nanoparticle	hPDLSCs	Rat model of ligature-induced periodontitis	promote osteogenic differentiation, reduce bone absorption by decreasing inflammatory infiltration and inhibiting osteoclast formation	Zhou et al., 2021 [78]
	Zeolitic imidazolate framework-8 nanoparticle loaded hydrophilic PVP, FK506 loaded PCL	nanofiber	Rat BMSCs	Rat model of ligature-induced periodontitis	exert antibacterial function benefiting the microenvironment for the osteogenesis process	Sun et al., 2022 [51]
	Gelatin/nano-HA microsphere embedded with stromal cell-derived factor-1	nanoparticle	Human MSCs	Rat model of mandible alveolar bone defect	enhance the alveolar bone regeneration	Fang et al., 2019 [34]
	Superparamagnetic iron oxide nanoparticles loaded gelatin sponge	nanoparticle	N/A	Rat model of mandible incisor sockets	obtain good visibility on MRI and enhanced bone regeneration without using an external magnetic field.	Hu et al., 2018 [79]
	HA nanowires modified polylactic acid membrane	nanowire	N/A	Rat model of mansible bone defect	promote the expression of multiple bone-related markers and form more bones with higher quality	Han et al., 2018 [35]
	Alginate encapsulated minocycline-loaded nanocrystalline carbonated HA	nanoparticle	Murine femur osteoblast cells	Rat model of maxillary alveolar bone defect	inhibit the growth of Enterococcus faecalis with cyto-compatibility and osteo-conduction properties	Calasans-Maia et al., 2019 [36]
	Nanostructured carbonated HA/sodium alginate containing strontium microspheres	nanoparticle	N/A	Rat model of maxillary alveolar bone defect	form new bone	Carmo et al., 2018 [80]
	Enamel matrix derivatives-loaded chitosan nanospheres embedded poly(D,L-lactic acid)-doxycycline	nanofiber	Murine osteoblasts	Rat model of maxillary alveolar ridge defects	accelerate wound healing and facilitate osteogenesis	Ho et al., 2022 [81]
	MXene (Ti_3_ C_2_ T_x_),	nanoflake	hPDLCs	Rat model of maxillary periodontal fenestration defects	exhibit biocompatibility and induce osteogenic differentiation, form new bone and inhibit osteoclast with enhanced expression of β-catenin, RUNX2, HIF-1α	Cui et al., 2021 [82]
	BMP-2 plasmid DNA-loaded chitosan nanoparticles	nanoparticle	N/A	Rat model of muscle pouches and calvarial defects and beagle model of ligature-induced periodontitis	increase new bone formation in rat calvarial defects and enhance bone healing in beagle dog, with non-specific inflammation	Li et al., 2017 [65]
	Cerium oxide nanoparticles	nanofiber	hPDLSCs	Rat model of periodontal bone defects	promote hPDLSCs osteogenic differentiation and accelerate new bone formation	Ren et al., 2021 [83]
	Chitosan, nano-HA and amnion membrane	nanofiber	N/A	Patient with gingival recession defects	enhance bone growth while prevent the gingival tissue downgrowth	Dhawan et al., 2021 [37]
	PLGA nanoparticles, CS nanoparticles and silver nanoparticles	nanoparticle	hPDLCs	Rabbit model of mandible bone defects	have an optimal proportion, show no cytotoxicity and contribute to cell mineralization.	Xue et al., 2019 [49]

### 4.2. PDL Regeneration

The nanomaterials applied in PDL, cementum and periodontal complex regeneration are listed in Table 2. Three of the studies included in this review are for PDL and alveolar bone regeneration [38,70,84]. It is important to remark that they all shared several common limitations: firstly, the study design lacked GTR or gold-standard bone grafts as controls. Secondly, they all used an animal model of experimental bone defect, which could neither simulate a chronic inflammatory microenvironment nor reflect the regenerative effect of the nanomaterials in complex periodontal defects. Thirdly, the underlying mechanisms of PDL and bone regeneration were not fully investigated. Moreover, Zhang et al. [84] stated that large animals should be used for more robust experiments and a variety of AuNPs should be constructed for further investigations. El-Sayed et al. [70] achieved a significant increase in functional PDL length but failed to increase the alveolar bone height, which might attribute to the deficiencies of material properties coupled with the possible peptide degradation in situ. The persistence of the peptides in the wound site was possibly too short, but the degradation profile of the peptide was not examined over time.

The study of Li et al. [85] was excluded from this review as it did not report new-formed PDL or alveolar bone after the application of nanoparticles. However, it is worth to mention that it describes periodontal tissue healing and reduced root resorption after tooth autotransplantation by applying nuclear factor-κB -PLGA nanospheres. PDL and alveolar bone regeneration were not directly detected in this study but were implied by a decrease in the expression of inflammatory markers and an increase in growth factors in PDL.

PDL is a specialized connective tissue surrounding and attaching teeth to the alveolar bone. There are several specific challenges in PDL regeneration. Firstly, the space that the PDL occupies, which spans approximately 150–400 μm from the alveolar bone to the cementum, is extremely small and thus limits the possibilities of applying medication or large transplants [86]. Although nanomaterials may present advantages in this point, it is still very difficult to regenerate soft tissue between two mineralized surfaces and specifically anchor it to them, while respecting the PDL space [87]. Notably, the PDL periodically undergoes various combinations of mechanical loading (i.e., compression, stretch, fluid-induced shear stress), contributing to maintaining the homeostasis [88]. Natural PDL fibers have a self-repair mechanism to preserve their integrity. They are aligned according to the magnitude and direction of loading, which increases their mechanical strength in this direction [89]. Tissue engineering technology combining biomimetic scaffolds and stem cells aims to mimic the properties of natural PDL and have opened a range of new therapeutic strategies in periodontal regeneration. However, there is a great challenge in the functional alignment of engineered nanofibers.

Three-dimensional bioprinting techniques have been widely used in craniofacial tissue engineering [90] and three-dimensional-printed scaffolds can aid complex periodontal reconstruction [91]. Nevertheless, such techniques only control the external properties of the scaffolds and not their internal architecture. The distribution of the seeded cells has also been reported to be heterogeneous [92]. Electrospinning has also been suggested as an effective approach to regenerate PDL [93], as it can improve the orientation of collagen fibers and reconstruct directional fiber bundles [94]. Combining 3D bioprinting techniques and electrospinning may overcome the shortcomings described above and produce effective biomimetic nanomaterials for PDL regeneration.

**Table 2:** Nanomaterials used for PDL, cementum and periodontal complex regeneration.

Application	Nanomaterials	Morphology	*In Vitro* Experiments	*In Vivo* Experiments	Outcomes	References
Alveolar bone and PDL regeneration	Polycaprolactone (PCL), cross-linked alginate, nano-HA (HA)	nanofiber	Dog adipose-derived MSCs	Dog model of class II furcation defects	yield periodontal wound healing, type I collagen of newly formed bone and PDL, and enhance the expression of VEGF and osteopontin	Mansour et al., 2022 [38]
	L/D-cysteine-anchored AuNPs	nanoparticle	Human periodontal ligament cells (PDLCs)	Rat model of mandible periodontal fenestration defect	show a better performance in cellular internalization, autophagy regulation, osteogenic differentiation and periodontal tissue regeneration	Zhang et al., 2021 [84]
	Self-assembling peptide (SAP; P_11_-4)	nanoparticle	N/A	Rat model of maxillary periodontal defects	increase functional PDL length and reduce epithelial down growth after 4 weeks, with a significant increase in osteocalcin and OPG and higher OPG/RANKL ratio	El-Sayed et al., 2020 [70]
Alveolar bone and cementum regeneration	Chitosan, type I collagen, Poly(ethylene oxide), (Arg-Gly-Asp) peptide, acetic acid solution, HA nanoparticles	nanoparticle	Human dental pulp stem cells (DPSCs)	Nude mice, rat model of mandible periodontal defect, mini-swine model of mandible periodontal defect	facilitate the regeneration of dentin, cementum and alveolar bone	Yan et al., 2022 [39]
Cementum-ligament-bone complex regeneration	Intrafibrillarly mineralized collagen and unmineralized parallel-aligned fibrils	biphasic scaffold	hPDLSCs	Rat model of complete periodontal defect model	reconstruct native periodontium with the insertion of PDL fibers into newly formed cementum and alveolar bone by recruiting host MSCs	Yu et al., 2021 [43]
	ε-aminocaproic acid-releasing chitosan particles-incorporated fibrin	nanoparticle	Cementoblasts and MC3T3-E1	Beagle model of mandible Class II furcation defect	promote alveolar bone and cementum formation, develop structural integrations of the cementum-PDL-bone complex by the Sharpey’s fiber insertion,	Park et al., 2017 [95]
	Enamel matrix derivatives and bone morphogenetic protein-2 loaded biphasic cryogel scaffold	nanofiber	MSCs	Beagle model of mandibular periodontal intrabony defect	have potential for the reconstruction of alveolar ridge, PDL and cementum	Huang et al., 2020 [96]
	chitin–PLGA/nBGC/CEMP1, chitin–PLGA/FGF2 and chitin–PLGA/nBGC/PRP	nanocomposite	Human dental follicle stem cells	Rabbit model of maxillary periodontal defects	achieve simultaneous and complete periodontal regeneration	Sowmya et al., 2017 [47]
	15-deoxy-Δ12,14-prostaglandin J2, PCL/GE	nanoparticle	decellularized hPDLC sheets	Rat model of mandible periodontal fenestration defect	form new bone, cementum and PDL	Jiang et al., 2021 [52]
	Dimethyloxalylglycine, nanosilicate, PLGA	nanoplatelet	hPDLSCs	Rat model of mandibular buccal bone defect	promote the recruitment of CD90+/CD34− stromal cells, induce angiogenesis and osteogenesis and regenerate cementum-ligament-bone complex	Shang et al., 2021 [48]
	Gold nanoparticles	nanoparticle	hPDLCs and human macrophages	Rat models of both fenestration and ligature-induced periodontitis	increase newly formed periodontal attachment, bone and cementum in periodontal defect with less tissue breakdown in periodontitis	Ni et al., 2019 [60]

### 4.3. Cementum Regeneration

The tooth cementum is a calcified connective tissue covering the outer surface of the dental root, which provides a medium for attachment and insertion of PDL fibers. The cementum can become damaged or deteriorate over time, especially in cases of advanced periodontal disease or aggressive tooth brushing. This can lead to tooth sensitivity, root decay and other oral health problems. Treatment options for cementum loss may include scaling and root planning, gingival grafting and other procedures to restore the tooth and surrounding tissues.

Only one article in this review reported alveolar bone and cementum regeneration, which was based on the grafting of exogenous DPSCs embedded in an osteogenic scaffold [39]. This article uses a patch to deliver DPSCs with differentiation potential and paracrine functions to suppress the local inflammatory reaction. Moreover, this patch inhibited epithelial invasion, providing necessary space for periodontal regeneration and satisfying the requirements for GTR membranes. However, PDL regeneration was not mentioned in this study. The regeneration of cementum with PDL attachment remains difficult.

### 4.4. Regeneration of the Cementum-Ligament-Bone Complex

Seven of the included articles reported regeneration of the cementum–ligament–bone complex [43,47,48,52,60,95,96]. The periodontium exhibits a typical “sandwich structure” composed of the cementum, alveolar bone and PDL with fiber bundles in various directions. Recently, the key to periodontal regeneration by tissue engineering has become the reconstruction of polyphase scaffolds and different periodontal fiber orientations, mimicking the interaction between the layers and the distinctive alignment of periodontal fibers [97,98]. It is crucial to highlight that all of the aforementioned studies show that nanomaterials alone might not be the only solution for producing successful bone regenerative scaffolds. A hierarchical design would also be needed to build the optimal biomimetic nanomaterials. Eventually, it might be possible to develop an ideal scaffold by combining various components and methods [28].

Since the regeneration of the cementum–ligament–bone complex usually requires a hierarchical design with multiple components, the biomedical safety of the used formulations brings great concern. However, as those nanomaterials are still in the experimental stage, very little attention has been paid to this point, which is reviewed and discussed in the last chapter of this review, ‘Biomedical safety.’ In addition, the synchronized or well-organized biodegradation time among different components is also crucial to the function of the nanomaterials. A too-fast biodegradation of the scaffold may not provide enough mechanical strength or necessary time for drug release, while a too-slow biodegradation can induce a host reaction to the synthetic materials [52]. Finally, some other problems are still to be solved. For example, can the nanomaterials still promote complete regeneration when the periodontal defect exceeds the critical size? Can the nanomaterials be used in an environment of microbial contamination?

### 4.5. Gingiva Regeneration

The gingiva is part of the oral mucosa that extends in the form of a collar around the enamel–cement junction of the tooth and covers the alveolar extensions of the maxilla and mandible. It adheres to the enamel via the junctional epithelium and attaches to the cementum and the bone through the collagen fibers of the lamina. The interdental gingiva occupies the space between two adjacent teeth and its flexible shape adapts to this space [99]. Gingival recession is a mucogingival defect defined as the apical shifting of the gingival margin in relation to its physiological position, located 1–2 mm coronally to the cementoenamel junction (CEJ) [100]. Patient-contributed trauma and iatrogenic interventions, such as improper toothbrushing technique, deep cervical restorative margins and orthodontic tooth movement, have all been associated with gingival recession [101].

None of the studies included in this review used nanomaterials to promote gingival regeneration due to a lack of in vivo studies. However, there are two in vitro studies that might be useful for gingival tissue engineering [102,103]. The reasons for the lack of studies are probably as follows: Firstly, up until now, therapeutic approaches for gingival recession have been oriented towards periodontal plastic surgery for root coverage and CEJ reconstruction [100,104]. Secondly, unlike alveolar bone defects, which can be covered and protected by gingiva after applying the nanomaterial-based transplant, the gingival wound or recessive gingiva itself is directly exposed to the complex microenvironment of the oral cavity. Third, applying nanomaterials may change the color, shape and texture of the gingiva, which can greatly influence smile aesthetics, especially when it happens in anterior teeth.

### 4.6. Biomedical Safety

Toxicity is an important consideration for the biomedical applications of nanomaterials and nanotechnology. Due to their extensive interactions with biological environments following their in vivo administration, nanomaterials can potentially have different degrees of toxicity to the body. First, nanoparticles interact with the blood cells and components once they enter the bloodstream, which can lead to hematological toxicity. Second, extensive amounts of nanoparticles can be accumulated in organs, especially those of the reticular endothelial system, which can result in toxicity to specific organs, such as the lungs, liver, spleen, kidneys or induce endocrine and immunotoxicity. Most of the intracellular and in vivo toxicity induced by nanoparticles arises from the production of excess reactive oxygen species (ROS). High ROS levels can damage cells by peroxidizing lipids, altering proteins, disrupting DNA, interfering with signaling functions and modulating gene transcription. These can finally result in cancer, neurodegeneration, cardiovascular, renal or pulmonary disease [105].

The toxicity of nanomaterials has been largely decreased with the application of biodegradable materials [106]. However, it needs to be noted that not all biodegradable materials are deemed safe for application in humans. Thus, to avoid misuse of nanomaterials, the main aspects involved in their toxicity, including their size, shape and surface chemistry [15], are summarized in this review as follows:

The size directly determines the surface area of nanomaterials available to interact with biological environments. Therefore, size is a critical physicochemical property influencing the cellular response and in vivo fate of nanomaterials [107]. The cellular response includes cytotoxicity, penetration of the biological barrier and immune response. For example, nanomaterials of smaller size have a higher ratio of surface area to volume, which leads to an increase in their reactivity [108]. Additionally, a decrease in the size is known to positively affect their vascular permeation [109]. A smaller size seems to result in a longer circulation time and reduced accumulation in the liver and spleen [110]. Particularly, small nanoparticles are even able to pass through the blood-brain barrier. Thus, their toxicity must be taken into full consideration and the potential beneficial function of any new nanomaterials must always be weighed against the risk prior to their clinical application.

The shape is also a pivotal physicochemical property of nanomaterials that largely determines their in vivo fate, including macrophage uptake, blood circulation and biodistribution, margination, extravasation and disease targeting [107]. Spherical particles have been more extensively studied in circulation due to their simple geometry. For example, the drug delivery efficiency was significantly higher in spherical particles than rod and elliptical disks [111]. However, these particles are often associated with filtration into off-target organs. For instance, the slits in the spleen that allow for blood filtration are asymmetrical, which makes it difficult for spherical particles to pass through [112]. Other asymmetric nanoparticles have been observed to offer benefits in bloodstream circulation, target tissue penetration and drug release [113]. Nanostructures of disks, large compound vesicles and staggered lamellae exhibited a significantly more extended circulation than that of spheres, while staggered lamellae and disks had longer circulation half-life than large compound vesicles and spheres [114]. Interestingly, smart nanoparticles have been developed recently with shape-switching and adjustable stiffness properties [115]. At first, the rod-like profile of nanoparticles prolongs blood circulation time and enhances extravasation into tumor tissues. When exposed to the acidic tumor microenvironment, nanoparticles decompose to transform into a spherical shape with higher uptake and cytotoxicity to breast cancer cells. Such shape and stiffness-adjustable design might be prospectively applied in periodontal regeneration to acquire higher drug accumulation in situ, providing a stronger engineering scaffold and alleviating systemic toxicities to the blood, liver, kidney and heart tissues.

The surface properties of nanoparticles, such as charge, hydrophobicity and targeting ligands, have a significant impact on their ability to circulate and subsequently be internalized by cells [107]. Surfaces with net positive charges or grafting with targeting ligand/peptide promote binding with negatively charged cell membranes and thus enhance cellular uptake. In contrast, surfaces with neutral or negatively charges promote longer-term circulation but typically have lower internalization potential [112,116]. Stimuli-responsive nanoparticles with switchable negative/positive charges or hidden/exposed target ligands might prevent off-target interactions during circulation while promoting immobilization and cell uptake at the site of the endogenous or exogenously applied stimulus. For example, the tunable surface charge of lipid nanoparticles contributes to form stabilization in the physiological environment and enhances internalization/drug release in the slightly acidic tumor microenvironment, respectively [117]. Furthermore, surface hydrophobicity can also influence the internalization of nanoparticles. For example, the translocation abilities of hydrophilic nanoparticles can be enhanced by increasing their stiffness, while the penetrability of hydrophobic nanoparticles is weakened by increasing their stiffness [118]. These strategies of well-designed surface chemistry could also be utilized to inspire specific targeting of periodontal tissue regeneration.

## 5. Conclusive Remarks

The use of nanomaterials for periodontal tissue regeneration is still in the experimental stage. Further research is needed to optimize the design and application of nanomaterials and enhance their biomedical safety, especially for the use of (large) animal models mimicking chronic inflammation and testing the regeneration of cementum–ligament–bone complex. In spite of this, they have shown the potential to significantly improve the outcomes of periodontal tissue regeneration.

## Data Availability

No new data were created or analyzed in this study. Data sharing is not applicable to this article.
